# Hitler’s Jewish Physicians

**DOI:** 10.5041/RMMJ.10157

**Published:** 2014-07-25

**Authors:** George M. Weisz

**Affiliations:** School of Humanities, University of New England, Armidale, Australia and University of New South Wales, Sydney, Australia

**Keywords:** Hitler, hysterical blindness, Jewish physicians

## Abstract

The mystery behind the behavior of infamous personalities leaves many open questions, particularly when related to the practice of medicine. This paper takes a brief look at two Jewish physicians who played memorable roles in the life of Adolf Hitler.

## INTRODUCTION

History reveals that over the centuries Jewish people have been excluded from some professions, while being forced into specific ones. This led to them often finding a place in medicine and finance. Restricted to specific localities and work conditions, Jewish physicians nonetheless found their work appreciated even by perpetrators of anti-Semitism. Royalty and national leaders often engaged the services of private Jewish physicians, while officially supporting anti-Semitism. The list of famous personalities using the services of Jewish physicians is long. Of particular surprise is the connection two Jewish physicians had (albeit an inadvertent one) with the greatest persecutor of the Jewish nation, the Fuehrer of the Third Reich. Here are their stories.

## DR EDUARD BLOCH

**1908**: August–December, Linz, Austria. *The Hitler family send you the best wishes for a Happy New Year, in everlasting thankfulness. AH*.^1^

Dr Eduard Bloch was a general physician, practicing on the main street of the poor neighborhood of Austria’s third largest city, Linz. A promise was given to this Jewish doctor by a grateful patient: “I shall be grateful to you forever, A.H.,” followed by a postcard sent from Vienna.

**Figure 1.**
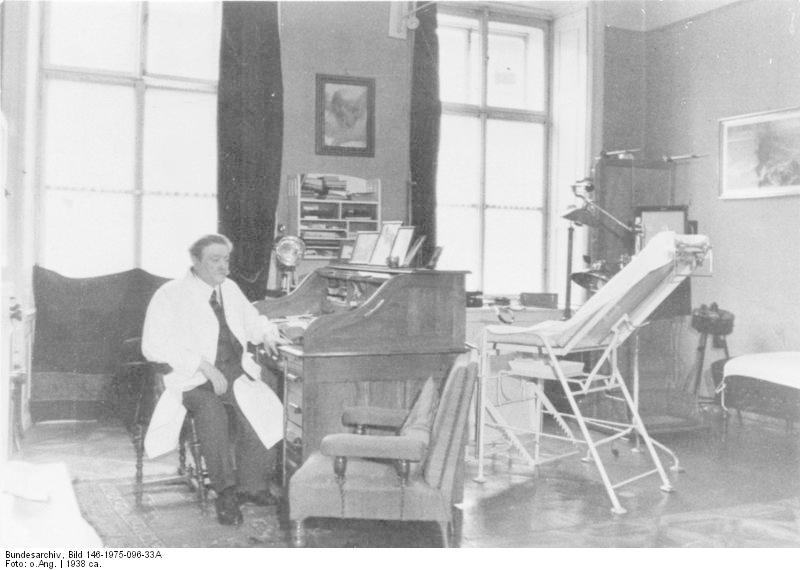
**Dr Eduard Bloch, Chief Medical Director of Health (GP Hitler family), in his consulting room in Linz.** From the German Federal Archive, Bild 146-1975-096-33A/O. Ang., reused under the Creative Commons Attribution-Share Alike 3.0 Germany license.

Eduard Bloch was born in 1869 to a Jewish family in Frauenburg, a small southern Bohemian village. He studied medicine in Prague, enlisted in the army of the Habsburg Empire, and was sent to Linz. After his discharge from the army he decided to settle in Linz, where he practiced for 37 years, serving the underprivileged and earning the title of “the poor man’s doctor” (Figure 1). He charged patients according to their financial status; he often took nothing at all.

In this capacity, in 1901, Dr Bloch came to look after the family of Alois Schicklgruber. Later, the name Schicklgruber would be changed to a variant of Hudler or Hutler, a name from the family of Adolf Hitler’s paternal grandmother.^2^

Dr Bloch cared for Alois’s first wife and two daughters and for his second wife Klara, the mother of Adolf and his sister Klara Jr. The illnesses treated were mainly children’s diseases, with four siblings succumbing to sickness—a statistic not uncommon for that time.

Adolf was born in 1889 in Braunau, a small border town between Germany and Austria. Later on, the family moved to Linz. The boy left school at the age of 16 and lived in Vienna. Twice he attempted to obtain acceptance in the Academy of Arts. He was rejected but was advised to study architecture, a topic more suitable for his talents. This advice was not heeded, and Adolf remained a wanderer in Vienna, surviving by painting postcards, and supported by a Jewish friend, the artist Joseph Neumann (later on called “a very decent man”).^3^

This bohemian lifestyle was interrupted in early 1907 when he was summoned to Linz; his beloved mother had been diagnosed with breast cancer of the ulcerated and possibly scirrhous type. The young son, always neatly dressed and quite courteous, was distraught over the suffering of his mother, and even more so later on following a clearly unsuccessful surgery. The extent of surgery performed is unknown; we do know that Halsted’s radical mastectomy was practiced at that time.^1^ Postoperatively, Klara was treated with the then commonly used local iodine compresses on the breast, this to “clean the malignant tissue,” an odorous and painful therapy.^4^

After protracted suffering, Klara died in December of 1907. A few days after the funeral Adolf and his three surviving sisters came to thank Dr Bloch for the help he had given to the family. Their gratitude was expressed repeatedly over the years. Indeed, back in Vienna, Adolf sent a congratulatory New Year’s postcard two years in a row. Years would pass until their life-paths would—figuratively—cross again.

### 1937, Berlin

**Figure 2.**
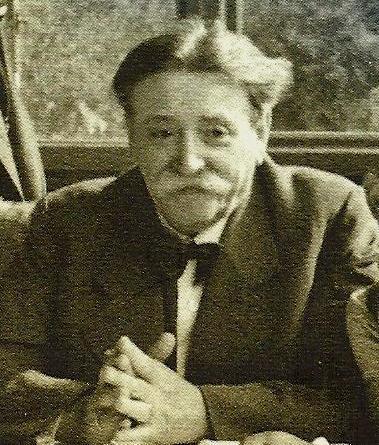
**Dr Eduard Bloch.** Taken from http://www.lidovky.cz/foto.aspx?r=ln_kultura&foto1=WOK47d27a_kul4hitleer.jpg; public domain.

The Fuehrer received Nazi delegates from Austria. He inquired about Linz and about Dr Bloch— whether he was alive and, if so, if he was still practicing medicine. The Fuehrer stated that Dr Bloch was a noble Jew, (“*Edeljude*”), further stating that “if all Jews would be like Dr. Bloch, there would be no Jewish question”^1^ (Figure 2).

### 1938, March, Linz

The German Army entered the Eastern province (Österreich), recently annexed to the Third Empire. The Fuehrer was in an open car traversing the main street in Linz. Whilst looking up to the old building of Dr Bloch, he made eye contact with the old physician, who was observing the parade from an upper window.

### 1939–45

Dr Bloch and his family were given special privileges that were probably not accorded to any other Jews in the Reich. Dr Bloch wrote (in his review dated 1941 in New York^1^) that two Gestapo officers came to his flat, requesting that he return several of the postcards that Adolf had sent to him in the past. The request was for *“*safekeeping the cards,” and a receipt was duly issued for them. The Blochs were allowed to keep their passports and their money; they were even finally able to withdraw their funds from the bank. Eventually, Dr Bloch was allowed to emigrate to the US (together with his wife, daughter, and son-in-law, also a physician).

In New York, Dr Bloch wrote in his review that during his entire career, he never saw a more distraught person than the young Adolf upon the death of his mother. He recalled asking himself “could this gentle boy be the Fuehrer?” He also asked: “What does a doctor think when he sees one of his patients grow into the persecutor of his race?”^1^

Dr Bloch lived in the Bronx until 1945. Before he succumbed to gastric carcinoma, he declared “I am 100% Jewish.” He was buried in the local Jewish cemetery.

## DR KARL KRONER

**1918:** October–November, Pasewalk, Pomerania, East Germany.

Ten years after first encountering Dr Bloch, Corporal Adolf Hitler, the future Fuehrer, would cross life-paths with another Jewish physician. Dr Karl Kroner was born to a medical family in Berlin, in 1878. He studied medicine locally and practiced in several hospitals. He also worked for the Siemens Health Insurance Group until 1920, when he started private practice. Once the Nazis took power, his practice was limited, but maintained in the Moabite (Jewish) Hospital.

The meeting between Adolf and Dr Kroner took place in October 1918 in a lazarett, namely a military hospital in Pasewalk on the Baltic shore, close to Stettin (Szczecin) on the Polish border. This psychiatric hospital was one of several lazarett units in the country and was established to treat shell-shocked soldiers.

### Backflash to 1907–8

Adolf, the teenager, had left Linz after the death of his mother and wandered for three more years in Vienna. He educated himself in German history, in nationalism, and in the Teutonic topics of Wagner’s music. Soon he ended up in Munich, in contact with the pan-German nationalistic movements. One might recall that he was born in a border town between Austria and Germany, and that he did not accept the division of the two countries or the separation of two peoples speaking the same language.

Adolf was elated at the prospect of war, and soon after the start of the Great War he joined in, becoming a messenger, running between units at great risk. He was wounded in the leg, for which he was decorated, but soon returned to his unit.

In October 1918, Adolf’s unit of over 100 soldiers was exposed to a gas attack whilst fighting the British at Wervicq-Sud (in the Pas-de Calais region of Northern French mountains), near Ypres on the Belgian border. The soldiers were treated locally in a makeshift hospital, with lavage of their inflamed conjunctiva. All were soon discharged except one, Adolf, who because of “blindness” was transferred to Pasewalk Hospital.

The British gas was most likely of the white phosgene type. It was characterized by conjunctival and nasal mucosal irritation; only in larger quantities did it produce respiratory mucosal irritation. It had an immediate effect, but was of a relatively short duration. In contrast, German mustard gas, the product used since 1915, was different. Indeed, it required hours or up to 2 days to affect the mucosa of the eyes and nose and had to be absorbed in larger quantities for there to be any lasting respiratory difficulties.

Loss of vision would have had to be the effect of either closure of the eyelids from swelling—a temporary reaction—or of corneal scarring, which took time to develop. Hence, it was suspected that Adolf’s immediate loss of vision was not organic in nature and required referral to a psychiatric hospital. The diagnosis was made locally in the nearby Oudenaarde Hospital in Belgium, and Adolf was sent to Ghent on his way to Pasewalk.

On admission to hospital in Pasewalk, Dr Karl Kroner, a Jewish neurologist (his picture can be viewed at http://www.dredmundforster.info/dr-karl-kroner), confirmed within minutes the diagnosis of “hysterical amblyopia,*”* one of the many forms of a reaction to trauma.^5^ Unlike the amblyopia, the chemical conjunctivitis cleared within days.

The diagnosis was further documented by a well-known German neuropsychiatrist Dr Edmund Forster, Chief of the Berlin University Nerve Clinic, who took over Adolf’s management from that time on.^6^–^8^

Traumatized patients at that time were treated differently. First they were criticized for their shameful and unmanly behavior. They were then treated aggressively with beatings, electro-shock, and various other punishments, all to bolster their self-esteem and promote a quick return to their unit. Another form of treatment was the “enlightened technique,” namely hypnosis. This is the technique that Dr Forster used on Adolf for his blindness: “it was to awake the patriotic duty in a future leader of a country.”^7^–^9^ Grasping the implanted idea, Adolf’s vision began to return.

A second relapse of Adolf’s “blindness” occurred in November 1918 when news of Germany’s capitulation arrived. It led to days of sobbing and moaning, and to sudden awakenings in screams converging into anti-Semitic rages. Hypnosis created in him the idea of a “divine mandate” for him to revive, lead, and revenge the humiliation that Germany had suffered. Later on, Adolf would write that this event brought about his decision to enter politics, a statement questioned by some historians.^2^,^9^–^12^

### 1933

On the ascendance of Adolf to Chancellorship of the Reich in 1933, the psychiatric hospital documents and the people involved had to be erased. Dr Forster was “interrogated” by the Gestapo for days. He was then interned in Dachau, but soon after was released. He returned to his family, only to commit suicide a day later with a pistol, although he was not known to have owned one.^9^ All the hospital documents “apparently” vanished.

### 1938

Dr Kroner, however, was in possession of the sole copy of hospital notes on Adolf. He forwarded them to a Jewish refugee surgeon-turned-writer, Dr Ernst Weiss in Paris.^13^ Tragically, Dr Weiss gave in to fear when the Nazis occupied Paris. In 1940, he committed suicide; unknown to him, Eleanor Roosevelt had already assured him an entry visa to the USA.^14^

Further quotes from the Pasewalk hospital notes were finally located in the Archives of the US Naval Service (disclosed only in 1972, the OSS Strategic Service).^15^,^16^

Dr Kroner was arrested in December of 1938, on Kristallnacht. He was deported to Sachsenhausen concentration camp, but was soon released, as his non-Jewish wife had obtained an entry visa to Iceland. He further emigrated to New York in 1945, where he lived until his death in 1954. He is buried in Iceland with his wife, whilst his son, Klaus, remained in the United States, practicing medicine.

## CONCLUSION

The history of these two physicians who looked after the young man, Adolf Hitler, first in his teens and then as a corporal in his twenties, is of interest more because Hitler, the future Chancellor of Germany, was the most rabid of anti-Jewish leaders in the world. Nevertheless he allowed both of his Jewish physicians to leave the Reich, whilst millions of others were murdered. Was this out of respect for the persons or for their profession, or perhaps a combination of both?
